# Frequency-domain ultrafast passive logic: NOT and XNOR gates

**DOI:** 10.1038/s41467-020-19544-9

**Published:** 2020-11-17

**Authors:** Reza Maram, James van Howe, Deming Kong, Francesco Da Ros, Pengyu Guan, Michael Galili, Roberto Morandotti, Leif Katsuo Oxenløwe, José Azaña

**Affiliations:** 1 Institut National de la Recherche Scientifique (INRS) – Energie, Matériaux et Télécommunications, Montréal, QC H5A 1K6 Canada; 2 Fonex Data Systems Inc., Montréal, QC H4S 1P6 Canada; 3 Department of Physics and Astronomy, Augustana College, Rock Island, IL 61201 USA; 4 Department of Photonics Engineering, Technical University of Denmark, 2800 Lyngby, Denmark; 5 State Key Laboratory of Information Photonics and Optical Communications, Beijing University of Posts and Telecommunications, 100876 Beijing, China

**Keywords:** Electrical and electronic engineering, Ultrafast photonics, Photonic devices, Information theory and computation

## Abstract

Electronic Boolean logic gates, the foundation of current computation and digital information processing, are reaching final limits in processing power. The primary obstacle is energy consumption which becomes impractically large, > 0.1 fJ/bit per gate, for signal speeds just over several GHz. Unfortunately, current solutions offer either high-speed operation or low-energy consumption. We propose a design for Boolean logic that can achieve both simultaneously (high speed and low consumption), here demonstrated for NOT and XNOR gates. Our method works by passively modifying the phase relationships among the different frequencies of an input data signal to redistribute its energy into the desired logical output pattern. We experimentally demonstrate a passive NOT gate with an energy dissipation of ~1 fJ/bit at 640 Gb/s and use it as a building block for an XNOR gate. This approach is applicable to any system that can propagate coherent waves, such as electromagnetic, acoustic, plasmonic, mechanical, or quantum.

## Abstract (web summary)

Typically, Boolean logic gates have to compromise between high speed and low energy consumption which can become limiting at scale. Here, the authors demonstrate architectures for NOT and XNOR gates that enable simultaneous low power and fast operation.

## Introduction

Boolean logic gates are the building blocks of computation and digital information processing. However, current logic gates, based on electronic transistors, are reaching physical device limitations^1–5^. Unless fundamentally new technologies are developed, growth in computational processing power, which has increased exponentially over the past 50 years, may come to a halt within the next two decades. The primary obstacle is the impractical amount of energy consumed while switching transistors from one logic state to another at high speeds^2,3^. Alternatives to electronic gates have been proposed using optics^6–8^, plasmonics^9–12^, magnetism^13,14^, phononics^15,16^, and electromechanics^17^. Such gates operate by nonlinearity, similarly to an electronic transistor, or by splitting/combining the signal in linear interferometric devices. However, all proposed alternatives to date have yet to show comparable, let alone superior, performance to current electronic logic gates. They are either too slow or too energy inefficient, and in some cases, they are based on unrealistic designs.

Recent experimental work and toy models show gates operating at or near the Landauer Limit^17,18^ (six orders of magnitude less energy consumption than current electronic complementary metal–oxide semiconductor (CMOS) gates); however, these approaches pay their energy savings with decreased speed (five to ten orders of magnitude slower). More typical gate designs, which use nonlinearity, have shown Boolean logic operations at ultrafast speeds, however, at the price of high energy consumption^19,20^.

Caulfield et al., who coined the term “zero-energy” logic, devised many theoretical designs that they claimed could operate at high speed and consume no energy by using passive, linear interference^21,22^. However, closer investigation showed that such gates were in fact energy hungry, and among other practical disadvantages, too physically large for realistic implementation^23,24^. Of many gate designs purporting to be “zero-energy,” “linear,” or “passive,” it is noteworthy that very few are entirely passive, and all consume some amount of energy.

Recently, truly passive Boolean logic gates have been demonstrated using linear interference by coherent perfect absorption^9^ and surface plasmon polaritons (SPP)^10–12^ in waveguide interferometers. Though promising, like previous attempts at gate design, these works have yet to show experimental demonstration of simultaneous low-energy consumption and high-speed operation. Though both methods have potential to achieve high-speed operation, speeds have only been shown up to ~1 kHz in proof-of-concept experiments.

In this work, we demonstrate a fundamentally different approach to ultrafast, all-passive logic gate design. Whereas previous methods for passive gates introduce phase shifts onto signal controls in the time-domain bit-by-bit for linear interference, our method imposes phase shifts in the frequency domain to spectral components of the signal, thereby processing the entire data stream at once. We show ultrafast, low-energy-consuming NOT and XNOR gate functions, which are directly applicable to all-optical shift registers, packet header processing, bit-error monitoring, modular arithmetic combinational and multi-valued logic, scrambling/ciphering, and binary-to-quaternary encoding/decoding^25–27^. The speed of our gates is determined by the bandwidth of the spectral phase filter, whereas energy consumption is only limited by the filter’s insertion loss, allowing for both simultaneous high-speed operation and low-energy consumption. In a proof-of-concept experiment, a NOT gate is demonstrated operating at a bit rate of 640 GB/s with an estimated energy dissipation of ~1 fJ/bit.

## Results

### Design of NOT and XNOR gates

**Fig. 1:**
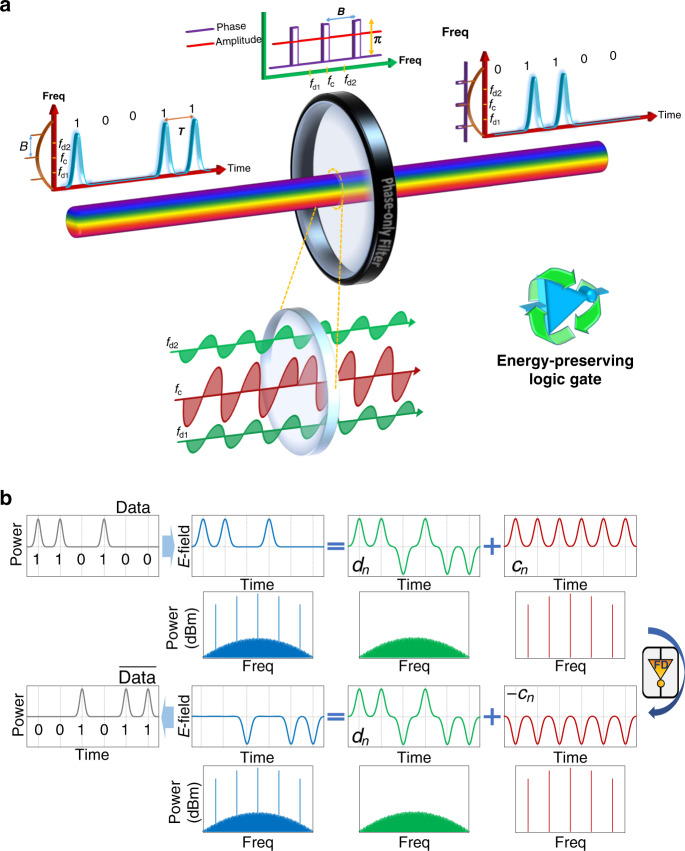
The operation principle of the proposed frequency-domain zero switching-energy logic NOT gate. **a** A frequency-domain (FD), passive NOT gate is implemented using a lossless passive all-pass (phase-only) linear filter. To perform the logical NOT operation on the digital (binary) input data signal, the filter imparts a *π* phase shift to the clock components of the incoming coherent digital input with respect to the rest of the signal frequency spectrum. Through this process, the output energy spectrum remains unchanged with respect to that of the input signal; however, in the time domain, the logic states of the input bit sequence are reversed. *f*_c_ (red) shows one of the frequency clock lines and *f*_d1_ and *f*_d2_ (green) represent arbitrary data frequencies. Note how *f*_c_ (red) is flipped in orientation at the output of the filter compared to *f*_d1_ and *f*_d2_. **b** Conceptual understanding of how amplitude-modulated data streams can be thought of as a sum of their clock, *c*_*n*_ (red), and data, *d*_*n*_ (green), components. The difference between Data and its inverse, $\overline {{\mathrm{Data}}}$, is a *π* phase shift (flipping in orientation) on clock frequency tones (red) with respect to the spectral data components (green). The summation of inverted clock frequencies to the original data results in the NOT operation.

The demonstrated logic gate designs are inspired by previous work on optical signal processors based on spectral phase-only filtering^28–30^. Figure 1 shows the operation principle of the fundamental NOT gate unit proposed herein. In the illustrated example, an input temporal bit sequence is encoded in a return-to-zero (RZ) on–off-keying (OOK) modulation data format, where a binary “1” is represented by a high signal, a “0” as a low signal, and the data stream always returns to the zero-signal level momentarily after each bit. The time spacing between consecutive bits, *T*, defines the bit period, which is inversely proportional to the bit rate of the data stream, *B* = 1/*T*. As shown in Fig. 1a, the frequency spectrum of the data signal consist of (i) a periodic set of strong components at discrete frequency locations that are spaced by the input bit rate *B* (i.e., the clock lines) and (ii) weaker components corresponding to the data that are distributed continuously along the signal spectrum between the frequency clock lines. To implement the logical NOT operation on the input digital signal, we selectively impart a phase shift of value *π* to just the frequency clock components of the signal using a phase-only, passive, linear filter. The clock frequencies are advanced in time compared to the remaining frequencies that represent the data, and the resulting interference pattern of all spectral components at the output is the exact reversed bit sequence of the input. Figure 1b shows a detailed explanation of how an amplitude-modulated signal can be composed of clock and data components in both the time and frequency domain, and how a phase-only spectral filter can implement the NOT gate operation.

The key to the conceptual understanding of the passive NOT gate is realizing that the electric field envelope of an amplitude-modulated data signal can be thought of as the sum of a clock signal, *c*_*n*_ (red), and a bipolar data component, *d*_*n*_ (green). “0-level” bits are simply the sum of negative data field amplitude pulses with positive amplitude clock pulses (which sum destructively), whereas “1-level” bits are the sum of positive data field amplitude pulses with the clock (which sum constructively). By inverting the amplitude of the clock, bit levels that previously destructively interfered will then constructively interfere, and vice versa, resulting in the target NOT operation. In this process, only manipulation of the phase, not the intensity, is employed so that the input signal energy is entirely reused to form the output logical pattern (except for practical insertion losses). Moreover, in contrast to previous NOT gate designs based on linear interference^12^, no additional energy other than the data stream itself is required for operation. A more detailed mathematical derivation for each step of Fig. 1b is shown in Supplementary Note 1. In the case of non-return-to-zero (NRZ) data signal, the clock signal in Fig. 1b is replaced with a flat line (envelope of the carrier) and the data signal does not return to zero for each bit period. To apply the NOT gate operation for NRZ data, one therefore simply applies the *π* phase shift to the central frequency tone (the carrier) instead of multiple clock tones.

**Fig. 2:**
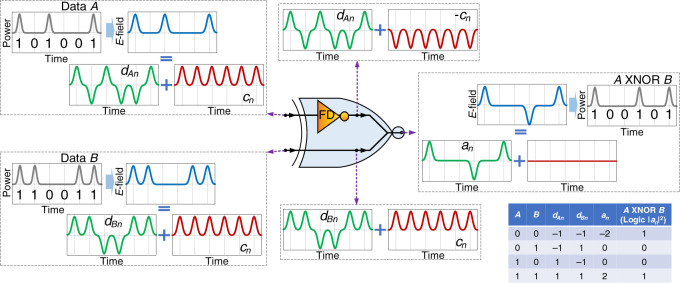
The operation principle of the proposed frequency-domain zero switching-energy logic XNOR gate. Conceptual illustration of how a frequency-domain (FD) passive NOT gate and a combiner can be used to implement an ultrafast passive XNOR operation. The FD NOT gate inverts the clock of Data *A* (red), which when summed with Data *B* at the combiner destructively cancels the clock of Data *B*. The resulting output field, *a*_*n*_, is simply the sum of the data field amplitude of Data *A*, *d*_*An*_, with the data field amplitude of Data *B*, *d*_*Bn*_, *a*_*n*_ = *d*_*An*_ + *d*_*Bn*_. The output intensity, |*a*_*n*_|^2^, gives the XNOR operation. The corresponding truth table is shown in the lower right-hand corner.

Figure 2 shows the operating principle of a passive ultrafast XNOR gate based on frequency-domain, passive NOT operation. Here the gate (i.e., a Y-branch waveguide for the case of optical signals) simply combines (i.e., sums) two input data streams with one of the inputs inverted by the frequency-domain, passive NOT gate. Whatever device is used as a combiner, it must be designed so that there is no phase difference between the signals at the combination point other than the inverted clock of one of the data signals (Data *A* in this example). When we apply data signals to the proposed XNOR gate, we also assume that the two data signals (Data *A* and Data *B*) have the same carrier frequency, same amplitude (for perfect operation), are coherent with each other, and have the same bit rate (which occurs, e.g., when Data *A* and Data *B* are derived from the same oscillator and the delay between data streams *A* and *B* is within the oscillator’s coherence time). The conceptual understanding of the frequency-domain passive XNOR gate is similar to that of the proposed NOT gate. We assume that the overall field amplitudes of Data *A* and Data *B* are each the sum of bipolar field amplitude data signals, *d*_*An*_ and *d*_*Bn*_, respectively (green), with their corresponding clocks, *c*_*n*_ (red). By inverting the clock of Data *A* (using a frequency-domain NOT gate) and summing it with Data *B* at the combiner, the clock signals of Data *A* and Data *B* destructively interfere, leaving a field amplitude output, *a*_*n*_, that is the sum of the input data field amplitude components, *a*_*n*_ = *d*_*An*_ + *d*_*Bn*_. The resulting output intensity |*a*_*n*_|^2^ gives then the target XNOR operation. A more detailed mathematical derivation for the XNOR gate can be found in Supplementary Note 2.

We emphasize that the NOT gate in the combiner in Fig. 2 is not a standard Boolean logic gate but rather the novel NOT gate design proposed herein. Such a schematic for the XNOR only works in this proposed implementation, which takes into account the phase of data and clock signals to perform logical operations, and should not be confused with a standard Boolean circuit diagram (i.e., in standard Boolean logic, ${\bar{{A}}} + {{B}} \,\ne\, {{A}}\,{\mathrm{XNOR}}\,{{B}}$). This gives the gate both advantages and disadvantages when compared to a standard Boolean XNOR device.

One practical advantage of the proposed XNOR is that our technique outputs true Boolean “1” and “0” in the output intensity, ideally giving infinite extinction between logic states when detecting power of the output data stream. This is in sharp contrast to previous attempts of passive logic that combine two Boolean inputs. For example, the interferometric gates shown by refs. ^10–12^, even under ideal conditions, produce multiple power-level outputs (i.e., the summation of data streams that represent an output “0” do not fully cancel to a zero level; likewise those data streams that represent a “1” do not constructively interfere to the same high-level output). Such multilevel output gates require lowering the threshold level that determines a “1” output and raising the threshold level that determines a “0” output in order to convert them into a true Boolean output. Unfortunately, this translates into the need for additional power-consuming nonlinear thresholding elements. Additionally, this also lowers the noise tolerance of the resulting gates and makes them much more prone to logic state errors. We note that for physical systems where the field/amplitude of the signal can be directly detected (such as electronic or acoustic) our XNOR gate produces bipolar outputs such that three levels are allowed: “−2,” “0,” and “2” (see the truth table in Fig. 2 and the related mathematical derivations in Supplementary Note 2). In this case, thresholding will be needed for binary operations, which can be simply obtained by detecting power (modulus squared of the amplitude) at the receiver or by rectifying the signal before detection. However, in some systems a bi-polar output may be desirable for denser information coding.

One disadvantage to the XNOR gate proposed here is that its sensitivity to the phase implies that two XNORs cannot simply be cascaded. Another is that the XNOR output is reduced by 3 dB (compared to the combined input) since energy of the clock signals from Data *A* and Data *B* destructively interfere at the combiner output (shed into the higher order modes). However, in a 2 × 2 combiner, this clock energy could be retained and reused for other operations downstream.

**Fig. 3:**
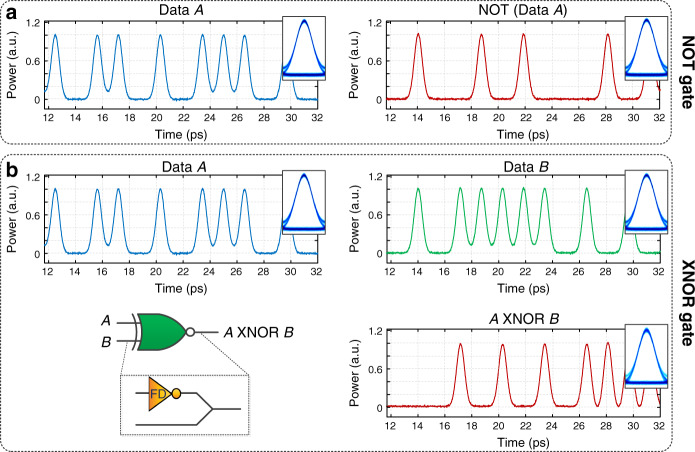
Demonstration of frequency-domain, passive logic NOT and XNOR gate through numerical simulation. **a** Left: 13 bits at 640 Gbit/s of a 128 random bit sequence (RBS) input to NOT gate, Right: NOT gate output of same 13 bits, insets: eye diagrams. **b** Top: 13 bits at 640 Gbit/s of two 128 random bit sequence inputs to the XNOR (Data *A* and Data *B*), Bottom right: corresponding XNOR output of 13 bits from logical operation of Data *A* and Data *B*, insets: eye diagrams.

To quantify gate performance, we numerically analyze the NOT and XNOR gates in Fig. 3, which shows the input time trace for 13 bits of a 128-bit random bit sequence (RBS), the output time trace of each gate for the corresponding 13-bit input(s) and the input and output eye diagrams (insets). The eye diagram is a graphical figure of merit for information processing systems and is generated by superimposing successive bit waveforms, in order to form a composite image of the signal. The amount of eye opening is related to the bit error rate (BER) and therefore system performance. A closed eye means the device or system between the transmitter and the receiver is distorting bit levels of the input signal so much that it introduces errors in the received data. In our simulation, “1” bits are coded by Gaussian pulses, each with a 400-fs full-width at half maximum (FWHM) time width, and the signal bit rate is chosen to be 640 Gbits/s. The input RBS has an even distribution of “1s” and “0s”, which is the most common practice to maximize the entropy (average information) per bit and to minimize the overall probability of bit error^31^. See Supplementary Fig. 1 for a more detailed analysis on gate performance for an uneven distribution of “1s” and “0s.”

Figure 3a shows the input and output for NOT gate operation. The simulated gate applies periodic *π* phase shifts to clock frequency components spaced by the input bit rate *B* = 640 GHz, where each phase shift occurs over a spectral linewidth of 8 GHz. The logic gate flips the state of the logical input signal (left) and generates the reversed (NOT) logical output (right) with negligible distortions on the temporal shape of the individual bits and eye diagram. Figure 3b shows simulated performance of the XNOR using the same parameters as the NOT gate in Fig. 3a. Like the NOT gate, the proposed XNOR performs the target logical operation with negligible distortions on the temporal shape of the individual bits as well as the eye diagram.

### Experimental demonstration of NOT gate

**Fig. 4:**
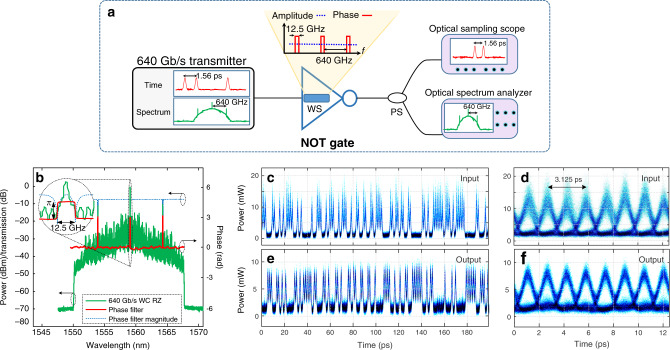
Proof-of-concept demonstration of the frequency-domain zero switching-energy logic NOT gate. **a** Schematic of the experimental set-up. Arrangement consisting of a 640-GHz optical RZ data transmitter, the logic NOT gate, and a receiver to measure the temporal waveform and spectrum of the output signal. The logic NOT gate is implemented using an optical Waveshaper (WS) programmed to produce the needed phase-only linear filtering specifications, i.e., a constant spectral amplitude and a spectral phase profile with *π* phase shifts periodically spaced by 640 GHz and with each shift extending over a line-width of ~12.5 GHz (determined by the minimum frequency resolution of the WS). **b** Spectra of the coherent data signal input to and output of the logic gate (they are overlapped), as well as spectral phase profile of the used phase filter (red). **c**, **e** Temporal traces of the 640 Gbit/s coherent digital input data signal (**c**) and the inverted output data signal (**e**, synchronized with respect to the input for representation purposes). **d**, **f** Eye diagram (overlapping the temporal signals over eight consecutive bit time periods) corresponding to the input and output data signals shown in plots **c** and **e**, respectively.

Next, we experimentally demonstrate the frequency-domain, passive logic NOT gate design at a bit rate of 640 Gbit/s. For this purpose, the input signal is generated at optical wavelengths around 1537 nm to take advantage of the speed offered by optics. Figure 4a shows a circuit schematic of the experimental set-up. The input data signal, the 640 Gbit/s RZ data pulses, each with a 720-fs FWHM time width, is generated by optical time division multiplexing (OTDM) of 64 × 10-Gbit/s OOK tributary data channels. Further details of the experimental set-up can be found in Supplementary Fig. 2 and the Methods section. The input signal is then delivered to the optical NOT gate unit. A commercial, programmable, spectral Waveshaper (WS) was used to produce the needed filtering. The spectral clock components of the input signal, spaced by 640 GHz, are phase-shifted by *π* with respect to the rest of the signal Fourier spectrum, as shown in Fig. 4b.

Figure 4c, e show the measured temporal intensity traces of the 640 Gbit/s coherent RZ input data signal and the output of the optical NOT gate, confirming inversion of the logical levels of the input bit sequence. Figure 4d, f report the corresponding eye diagrams of the input and output data signals, showing an observed improvement of the quality of the inverted data signal with respect to the input. This points out to a key additional feature of the proposed logic gate design, namely, its intrinsic logic-level restoration capabilities, as numerically demonstrated through the analysis shown in Supplementary Figs. 3 and 4. Qualitatively, we understand this restoration as an averaging effect of the phase-only filtering operation, similar to temporal Talbot noise mitigation^32–34^. We note that, because the “1” levels contain all of the fluctuation for amplitude and timing jitter, this noise from the input is transferred to the “0” levels but not the other way around. Thus, at the NOT gate output, the “1” levels become cleaner at expense of the “0” levels. However, due to an overall averaging, the net effect is to open the eye diagram and improve the output *Q*-factor (a figure of merit that quantifies eye opening, see Supplementary Fig. 3). A more detailed theoretical analysis and experimental investigation will be required to determine quantitatively how much the *Q*-factor can be improved for a given set-up, as well as the existence of any possible analytical trend.

**Fig. 5:**
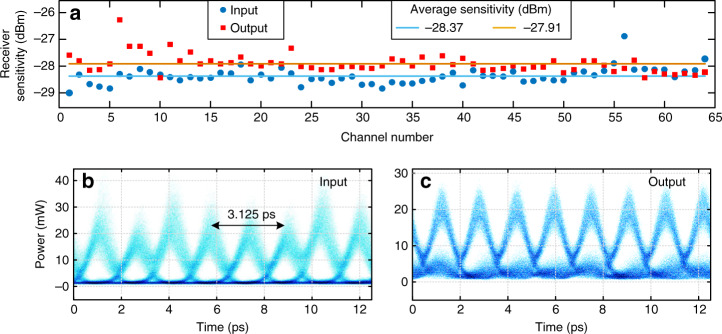
Experimental results. **a** Receiver sensitivity, i.e., required average power at the output of the detection system to ensure a bit error rate (BER) <10^−7^, measured for each of the 64 channels (at 10 Gbit/s) of the 640 Gbit/s OTDM input (blue) and output (red) signals. **b**, **c** Eye diagrams (overlapping the temporal signals over eight consecutive bit time periods) of the 640 Gbit/s coherent digital **b** input data signal and **c** the inverted output data signal.

BER measurements were carried out by first demultiplexing the 640 Gbit/s data sequence into 64 × 10-Gbit/s data signals using a nonlinear optical loop mirror (NOLM), details of which are provided in Supplementary Fig. 2 and the Methods section. Figure 5a shows the measured receiver sensitivity for all the 64 time-multiplexed channels of the input (blue circles) and output (red rectangles) data signals, considering a BER threshold of 10^−7^. Successful inversion of the input data, with a BER below this threshold, was achieved in all 64 channels for an average output signal power (at the receiver) above −27.91 dBm, even though the input data sequence exhibited notable intensity noise, see Fig. 4b, c.

The average receiver sensitivity is decreased only by ~0.46 dB from the input to the output of the NOT gate, from −28.37 to −27.91 dBm, with a maximum variation in the receiver sensitivity of the 64 channels below 2 dB. Note that the same 7.5 dB loss from the WS was incorporated for sensitivity measurements without the NOT gate so that reduction in receiver sensitivity (increase in BER) is attributed to the gate. This slight deterioration in the output BER as compared with the input is attributed to the minimum frequency resolution that could be practically implemented with the WS employed in the experiments, imposing a spectral linewidth larger than the optimal designed one. The effect of the spectral linewidth of the phase filter in the NOT gate operation is studied in depth in Supplementary Fig. 3c. In particular, simulation results in Supplementary Fig. 3c indicate that for a spectral linewidth of 12.5 GHz (the linewidth of the WS used in the experiments) we should indeed expect a slight BER deterioration, considering the lower quality of the eye diagram of the input data sequence employed in these specific experiments, Fig. 5b. Consistent with the numerical results shown in Supplementary Fig. 3, a restoration of the eye diagram from the input to the output would require either using a phase filter with a narrower spectral linewidth or improving the quality of the input data signal, as experimentally demonstrated in Fig. 4.

Additionally, although the coherence of the input signal is required for ideal operation of our logic gate technique, the signal output is negligibly distorted from the typical phase instability of off-the-shelf sources, even at a data rate of 640 Gbit/s. Supplementary Fig. 5 shows the output signal *Q*-factor after NOT gate operation using the same parameters as those in Fig. 3, as a function of source spectral linewidth. For a linewidth of ~10 MHz (coherence time of ~30 ns) the output *Q*-factor is >40. Degradation in the output signal quality does not become noticeable until the linewidth approaches ~300 MHz, giving an output *Q* = 5. Typical commercial optical telecom sources give spectral linewidths of 1–10 MHz while stabilized sources can reach <100 kHz, indicating that source coherence is not a limiting factor for successful gate operation. In general, for distortion-free output of our gate, the coherence time must be much longer than the duration of the data packet, here 128 bits/640 Gbs (or equivalently, the smallest frequency offset of the data tones to the clock, 640 Gbs/128 bits, needs to be much larger than the input signal spectral linewidth).

## Discussion

It is important to note that, although phase-only spectral filtering ideally preserves the entire input signal energy, practical filtering devices introduce losses that should be minimized to lower the energy consumption. In order to fairly calculate energy consumption of our device, we include all energy dissipated by passive losses. In our specific experiment, the average powers of the input and output data signals are ~5 and ~1 mW, respectively, considering that the total insertion loss of the phase filter is around 7.5 dB. This overall loss is due to the insertion loss of the WS device plus the loss that is induced through the use of additional amplitude filtering to ensure a flat spectral amplitude response across the entire signal frequency bandwidth. This translates into an estimated total energy loss per bit period of 6.4 fJ/bit, orders of magnitude lower than the energy that is consumed per bit period by previous designs working at a similar speed^20^. To compare to other devices, we use the approach by Tucker and Hinton^20^, which attempts to fairly measure energy consumption across different methods while normalizing for speed. In this calculation, our normalized energy consumption is 1.0 fJ/bit. For more details on our calculated energy consumption, see Energy consumption metrics in the Methods section. For comparison, the energy consumption for a simple nonlinear optical logic gate is ~100 fJ/bit, while CMOS gate energy consumption is ~0.1–1.0 fJ/bit (dominated by capacitive charging/discharging of the gate interconnect wires)^20,35,36^. However, practical clock speeds per core for CMOS technology have saturated in the range of 1–6 GHz^2^. Finally, the lowest energy consumption for a logic gate shown to date is ~0.1 aJ/bit^17,37^. Note, however, that such a low energy comes at a price of 1-kHz operation. Our results show record performance for low-energy operation in the ultrafast regime even though the specific phase filter used for demonstrations is not optimal for energy dissipation. The energy consumption of our gate could be further minimized by lowering the energy of the incoming signal or by reducing insertion losses in the linear optical filter through device and/or material optimization^38^.

In summary, this work introduces a novel, design concept for ultrafast logic gates based on passive, linear, phase-only filtering. Gate operation is fundamentally lossless and compatible with ultrahigh bit-rate implementations. We experimentally show NOT gate operation using optical signals at 640 Gbit/s and a relative 1.0 fJ/bit energy consumption. We further use our NOT gate to design an ultrafast, passive XNOR gate. Our concept is applicable to other physical systems (not just optical signals) that can propagate coherent waves. In terms of potential real-world device implementation, scaling the size of the phase filter will be critical. For optical signals, one could consider direct implementations based on integrated-waveguide Bragg gratings^39^ or through SPP generation in subwavelength waveguide structures^9–12^. Such circuits can be made with device lengths in the micrometer range or even smaller. More generally, there may be scaling advantages using our method with an entirely different physics. With further development, phase-only, frequency-domain filtering for logic gate design could offer solutions to energy consumption and speed limitations in present computation schemes.

## Methods

### OTDM signal generation and detection

We used a commercial Erbium-glass pulsed laser in the transmitter to generate optical pulses at 1542 nm with a pulsewidth of 1.5 ps and 10 GHz repetition rate. Subsequently, the pulses are compressed down to 720 fs by self-phase modulation in a 400-m-long section of dispersion-flattened highly nonlinear fiber (HNLF, from OFS), and filtered with an optical band-pass filter (OBPF) (Koshin Kogaku KD-2396) at 1537 nm with a 3-dB bandwidth of 16 nm, see Supplementary Fig. 2. The pulses are then intensity (OOK) modulated at the 10 Gbit/s base rate with a 2^7^ −1 pseudo RBS data. The modulated pulses are time-multiplexed in a passive fiber-based split-and-delay multiplexer (MUX) (Calmar BRM-T16) to constitute the 640 Gbit/s RZ OTDM signal. Next, the 640 Gbit/s RZ OTDM signal is subsequently wavelength converted using a polarization-rotating Kerr switch: the signal is first amplified using an Erbium-doped fiber amplifier (Keopsys EDFA) and coupled together with an amplified continuous-wave (CW) probe at 1559 nm in a 200-m HNLF (nonlinear coefficient: 10 W^−1^·km^−1^; zero dispersion wavelength: *λ*_0_ = 1552 nm; dispersion slope: *S* = 0.011 ps/(nm^2^·km)). At the fiber output, a polarizer is placed with its axis orthogonal to the CW light. The polarization of the data is 45° with respect to the polarizer. The CW light is blocked by the polarizer when the data signal is a “0” level bit. When the data signal is a “1” level bit, the Kerr effect in the fiber generates birefringence between the polarization direction of the data signal and its orthogonal direction. Therefore, in this case, the state of polarization of the CW light will rotate due to the induced birefringence, thus passing through the polarizer. As a result, the phase-uncorrelated OTDM data signal switches the CW light, and a pulse-to-pulse phase-correlated signal is generated at the CW light wavelength. Before wavelength conversion, an OBPF with bandwidth of 23 nm is used to filter out the amplified spontaneous emission introduced by the EDFAs. We emphasize that a coherent data signal is required for implementation of the proposed logic gate concept based on phase-only (all-pass) linear filtering.

### Phase-only filter

A line-by-line pulse shaper (Finisar Waveshaper 4000S) has been programmed to produce the needed linear filtering response, namely, a constant spectral amplitude response and a spectral phase response profile with *π* phase shifts periodically spaced by 640 GHz. The total loss of our filter consists of the device insertion loss of ~4.5 dB, plus an additional 3 dB of loss intentionally added to the data frequency components between the clock tones. The latter is necessary because the *π* phase step-function that is imposed at the clock tones inherently introduces a corresponding step-function of loss of ~3 dB at these components. This undesired additional spectral amplitude variation of the implemented filter is an intrinsic feature of the Waveshaper device that is used for practical realization of the desired filtering function (ideally, a phase-only spectral filtering function). We therefore need to induce this same value of loss (~3 dB) along the remaining frequencies to achieve the desired flat spectral amplitude response of the filter. The nominal minimum bandwidth (frequency resolution) of the Waveshaper is 10 GHz for implementation of amplitude filters and 12.5 GHz for implementation of phase filters. This latter specification determines the linewidth of the implemented phase shifts in our experiments. The spectral phase response of the phase filter was measured with a 1.6-pm spectral resolution Optical Vector Analyzer (Luna Technologies). It is worth noting that we use an active programmable Waveshaper to emulate the target passive filter, i.e., a filter with a prescribed fixed spectral response. While the programming of the filter induces additional loss, this is indeed not fundamental to the proposed concept. We note that, for data signals with different bit rates, the spectral spacing of the *π* phase shifts needs to be reprogrammed to match the clock repetition rate. As described, the logic operation is implemented by the passive, linear all-pass filtering process itself.

### Detection and BER measurement

All temporal measurements are carried out by a high-speed optical sampling oscilloscope (Exfo PSO-100) with a bandwidth of 500 GHz. The measurements of the power spectra of the signals involved in the reported experiments are carried out by an optical spectrum analyzer with a spectral resolution of 0.02 nm (Yokogawa AQ6370D). For the BER measurements, the 640-Gbit/s input and output signals are time-demultiplexed in a NOLM down to 10-Gbit/s signals. The NOLM operation is based on cross-phase modulation in a 50-m HNLF (zero-dispersion wavelength at 1545 nm, *S* = 0.015 ps/(nm^2^·km) at 1550 nm, and *γ* = 10.5 W^−1^·km^−1^) from OFS. The demultiplexed 10-Gbit/s signal is detected by a 10-Gbit/s optically pre-amplified receiver with a photodetector. The performance of the received signal is evaluated by a 10-Gbit/s error analyzer (Anritsu MP1800A). For the receiver sensitivity measurements reported in Fig. 5, we used a BER threshold of 10^−7^ rather than the standard BER threshold of 10^−9^; this was partly due to the relatively lower quality of the input data sequence that could be generated for proof-of-concept experiments, Fig. 5b, c.

### Energy consumption metrics

A fair comparison of energy consumption per bit across logic gate methods throughout literature is difficult at best. Authors often unintentionally underreport energy by not including amplifiers needed to boost the signal before the logic gate (power that is often needed for nonlinear optical gate operation) as well as fail to report energy consumed by active components in the gate that are continually powered, regardless of the presence of a signal^20^. We therefore use Tucker and Hinton’s work^20^ as a guide since they carefully and thoroughly analyze reported numbers from recent literature and add estimates of missing energy where authors have underreported numbers. In their comparison work, Tucker and Hinton note that, for optical approaches, energy dissipated per bit scales inversely proportional to clock speed. In order to compare reported energy consumption fairly, they normalize energy consumption of optical gates to a reference clock of 100 GHz.

In our specific experiment, the method for gate operation is entirely passive. Therefore, the energy dissipated per bit by our NOT gate is determined solely by the input power, loss, and speed of the gate. For our set-up, the insertion loss (IL) of the phase filter is IL = 7.5 dB (details provided above), which translates into an estimated total energy loss per bit period of $E_{{\rm{loss}}} = \left( {P_{{\rm{in}}} - P_{{\rm{out}}}} \right){\Delta}t = P_{{\rm{in}}}\left( {1 - 10^{ - {\rm{IL}}/10}} \right){\Delta}t = 6.4\,{\mathrm{fJ}}/{\mathrm{bit}}$, where *P*_in_ ≈ 5 mW and *P*_out_ ≈ 1 mW are the average powers of the input and output data signals, respectively, and Δ*t* ≈ 1.56 ps is the temporal bit period. Using 100 GHz as a reference clock to fairly compare our method to those in ref. ^20^, we find a normalized energy consumption of our gate to be $E_{{\rm{norm}}} = E_{{\rm{loss}}} \times (f_{{\rm{ref}}}/f_{{\rm{clock}}})$ = 6.4 fJ/bit × (100 GHz/640 GHz) = 1.0 fJ/bit. We compare this number to the energy consumption of single, simple optical and CMOS gates, which dissipate ~100 and ~0.1−1.0 fJ/bit, respectively^20,35,36^. To give more context, we also compare this to ultralow energy consumption shown by a recent electromechanical method^17,32^, which demonstrated a dissipation of ~100 k_B_T/bit, i.e., ~0.1 aJ/bit, but at a significantly reduced speed of ~1 KHz.

## Supplementary information

Supplementary Information

## Data Availability

The datasets generated during and/or analyzed during the current study are available from the corresponding author on reasonable request.

## Code availability

The computer simulation code used to report expected results, and compare experimental data to theory, is available from the corresponding author on reasonable request.
