# Supplementary material for: Frequency-domain ultrafast passive logic: NOT and XNOR gates
Source: Nat Commun. 2020 Nov 17;11:5839. doi: 10.1038/s41467-020-19544-9 (PMC7673018; doi:10.1038/s41467-020-19544-9)
Supplement: Supplementary file 1 — Supplementary Information [file 41467_2020_19544_MOESM1_ESM.pdf]

Frequency-domain Ultrafast Passive Logic: NOT and XNOR Gates

Reza Maram[1,4,†], James Van Howe[1,5,⁑], Deming Kong[2, 3], Francesco Da Ros[2], Pengyu Guan[2], Michael Galili[2], Roberto Morandotti[1], Leif Katsuo Oxenløwe[2] & José Azaña[1,*]

[1]Institut National de la Recherche Scientifique (INRS) – Energie, Matériaux et Télécommunications, Montréal, Québec, Canada, H5A 1K6, Tel.: +1 (514) 875 1266 – 7018 Fax: +1 (514) 875 0344.

[2]Department of Photonics Engineering, Technical University of Denmark, 2800 Kgs. Lyngby, Denmark.

[3]State Key Laboratory of Information Photonics and Optical Communications, Beijing University of Posts and Telecommunications, Beijing, 100876, China.

[4]Fonex Data Systems Inc., Montréal, Québec, Canada H4S 1P6.

[5]Department of Physics and Astronomy, Augustana College, Rock Island 61201, USA.

†⁑ These two authors contributed equally to this work.

*azana@emt.inrs.ca

Supplementary Information

Supplementary Notes (1, 2)

Supplementary Figures (1-5)

Supplementary Notes

Supplementary Note 1: Mathematical derivation for NOT operation. For an amplitude-modulated optical data stream, the electric field of a given arbitrary data signal, Data, may be presented mathematically by $e_a(t) = \sum_{n=-\infty}^{\infty} a_n u(t - nT)$, where T is the data clock period, $a_n = 0$ or 1 for OOK modulation, and $u(t)$ is the complex envelope of the optical pulse. This electric field can be decomposed into a superposition of the data and clock signals, namely, $e_a(t) = 0.5\{e_d(t) + e_c(t)\}$, where $e_d(t) = \sum_{n=-\infty}^{\infty} d_n u(t - nT)$ and $e_c(t) = \sum_{n=-\infty}^{\infty} c_n u(t - nT)$ represent the electric fields of the data and clock signals, respectively. For a data stream with binary intensity values 0 and 1, $a_n = 0.5(d_n + c_n)$, where $d_n = \pm 1$ and $c_n = 1$ for all *n*. The input signal intensity, $|a_n|^2$, equals 1 whenever $d_n = 1$ (i.e., $|a_n|^2 = |0.5(1+1)|^2 = 1$) and 0 whenever $d_n = -1$ (i.e., $|a_n|^2 = |0.5(-1+1)|^2 = 0$). Note that we assume that each bit has an equal probability of taking on the value “0” or “1” in the interval $[nT, (n+1)T]$.

One can achieve the desired $\overline{\text{Data}}$ by inverting the phase of the clock signal with respect to the spectral data components $(c_n \times e^{i\pi} = -c_n)$ or vice-versa$(d_n \times e^{i\pi} = -d_n)$. For experimental demonstration, we choose the former due to its simplicity. The output signal electric field coefficient will then be $a_n = 0.5(d_n - c_n)$. As illustrated in Fig. 1(b), the output signal intensity will be either (i) $|a_n|^2 = 0$, whenever $d_n = 1$, i.e., whenever the input bit is a logical “1”, $|a_n|^2 = |0.5(1-1)|^2 = 0$, or (ii) $|a_n|^2 = 1$, whenever $d_n = -1$, i.e., whenever the input bit is a logical “0”, $|a_n|^2 = |0.5(-1-1)|^2 = 1$.

Supplementary Note 2: Mathematical derivation for frequency-domain passive XNOR. Ensuring no additional phase between Data A and Data B from the combiner, and using the derivation above, the output of the XNOR can be described by, $a_n = 0.5\left(d_{An} - c_n + d_{Bn} + c_n\right)$ or simply, $a_n = 0.5\left(d_{An} + d_{Bn}\right)$. Recall that $d_n = -1$ for a logical "0" and $d_n = 1$ for a logical "1". The intensity output for the XNOR is therefore $\left|a_n\right|^2 = \left|0.5\left(d_{An} + d_{Bn}\right)\right|^2$.

Supplementary Figures

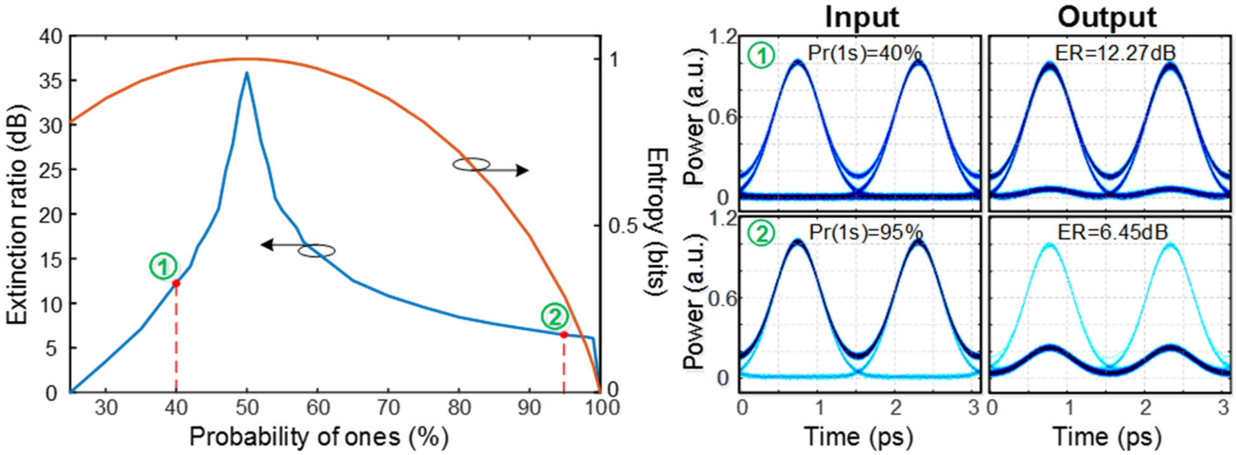

Supplementary Figure 1| Performance of NOT versus the distribution of "1s" and "0s" in the data sequence. Extinction ratio (ER), blue, of the output signal (the ratio of the "1" and "0" mean power levels in the eye-diagrams) and information entropy (red), $S = \sum_{i=1}^{2} -P_i \log(P_i)$, (where P_1 is the probability that the bit level is a "1" and $P_2 = (1 - P_1)$ is the probability that the bit level is a "0") versus the percent likelihood that the bit level is a "1" in the data sequence. The entropy shows the average amount of information in the system. Together the curves show that the best extinction ratio for the NOT operation occurs for maximum information content (typically the most useful operation point for an information processing system) and degrades for uneven distribution of "1" and "0" bit values. The insets to the right show the corresponding input and output eye diagrams for two simulation points of the ER graph.

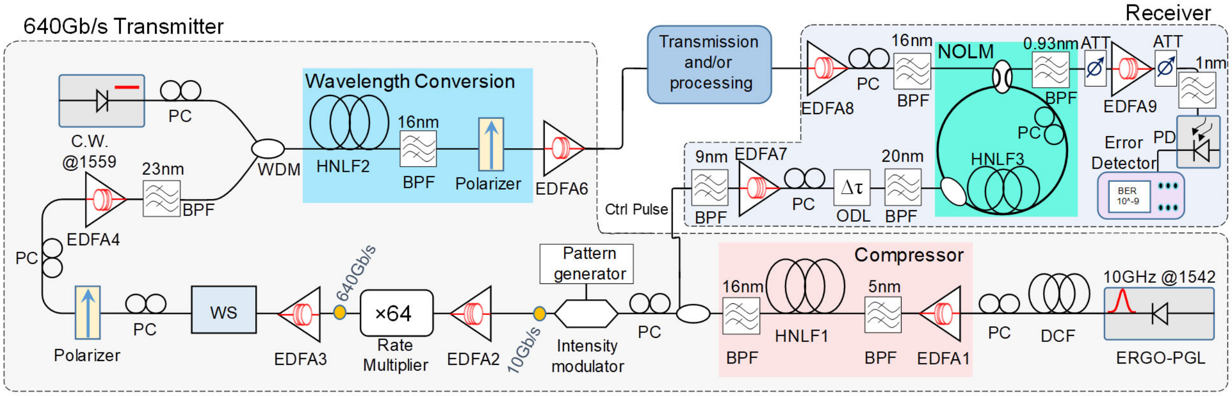

Supplementary Figure 2| Detailed experimental setup of the transmitter and the receiver for the BER measurements. In the transmitter, the input 640 Gbit/s RZ data pulses are generated by optical time division multiplexing (OTDM) of a 10-Gbit/s OOK data signal. The 640Gbit/s processed signal is demultiplexed in the receiver into 64×10-Gbit/s data signals using a NOLM for the BER measurements. ERGO- PGL: erbium-glass oscillating pulse generating laser; BPF: band-pass filter; DCF: dispersion compensating fiber; HNLF: high nonlinear fiber; PC: polarization controller; WS: WaveShaper; CP: 3dB coupler; EDFA: Erbium-doped fiber amplifier; C.W.: continuous wave laser; ODL: optical delay line; PD: photodetector; ATT: optical attenuator.

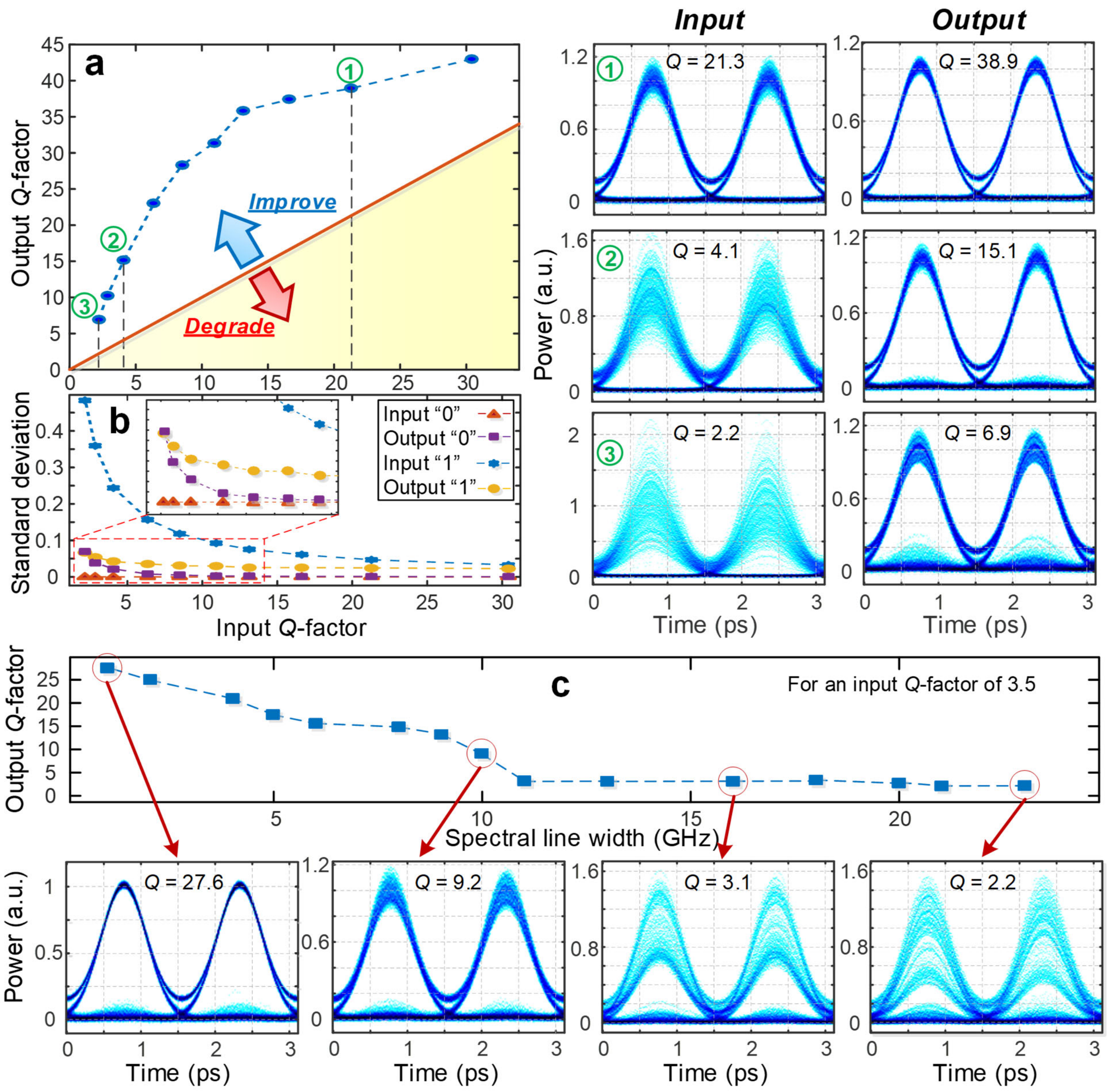

Supplementary Figure 3| Simulation results on the amplitude-fluctuations mitigation of an input binary data signal through linear NOT-gate filtering. The 640 Gbit/s 720 fs time-width RZ-OOK input signal, with 128 RBS data, is smeared due to amplitude fluctuation noise, modelled in the time domain with a random Gaussian distribution. **a** Output signal's quality factor (Q-factor) as a function of input signal's quality factor, showing significant improvement in the quality of the processed signal. The quality factor is defined as $Q=(P_1-P_o)/(\sigma_1+\sigma_0)$, where P_1 and P_0 are the

mean power values and σ_1 and σ_0 denote the standard deviations of the logic “1” and “0” levels, respectively. **b** Standard deviation of the power of “0” level and “1” level bits in the input and output signals. In the output signal, the standard deviation of the power for the “0” levels grows significantly faster than the “1” levels as the quality factor of the input signal reduces below 10. **c** Output signal’s quality-factor versus the spectral linewidth of the phase filter. The insets show some of the corresponding eye diagrams.

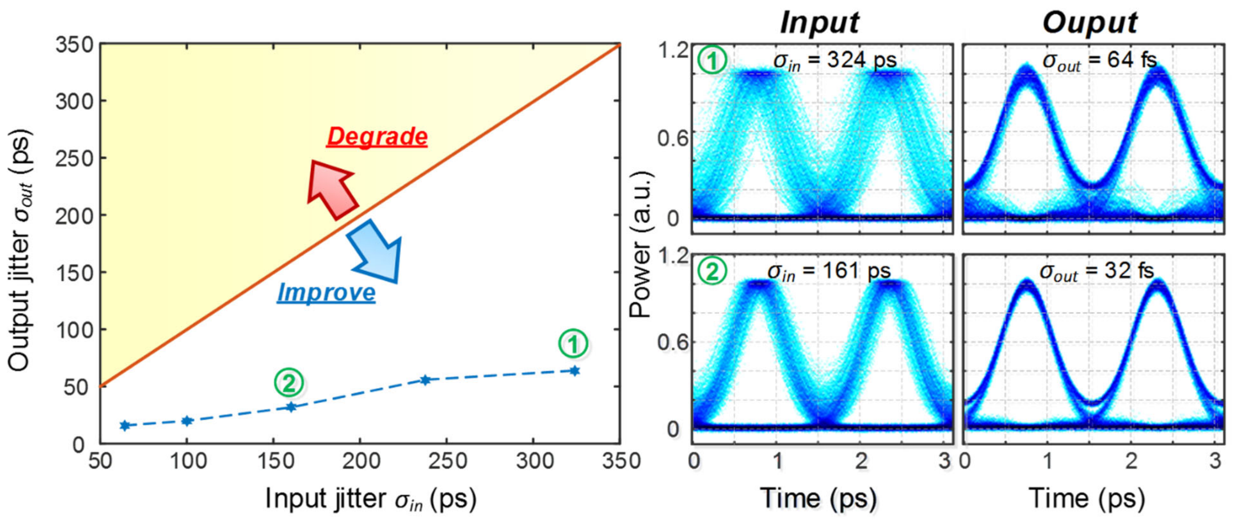

Supplementary Figure 4| Simulation results on timing jitter mitigation of an input binary data signal through the linear NOT gate filtering. Output jitter as a function of input jitter. The input timing jitter is modeled with a random Gaussian distribution. The standard deviation of the emulated timing jitter of the input signal varies from 80 fs to 330 fs and the timing jitter in the output is reduced to the range of 20 fs to 65 fs; however, deterioration on the pedestal of the output signal is also observed. It should be noted that previous logic schemes are unable to process such a jittery digital input because a stringent synchronization between the input and other signals is required.

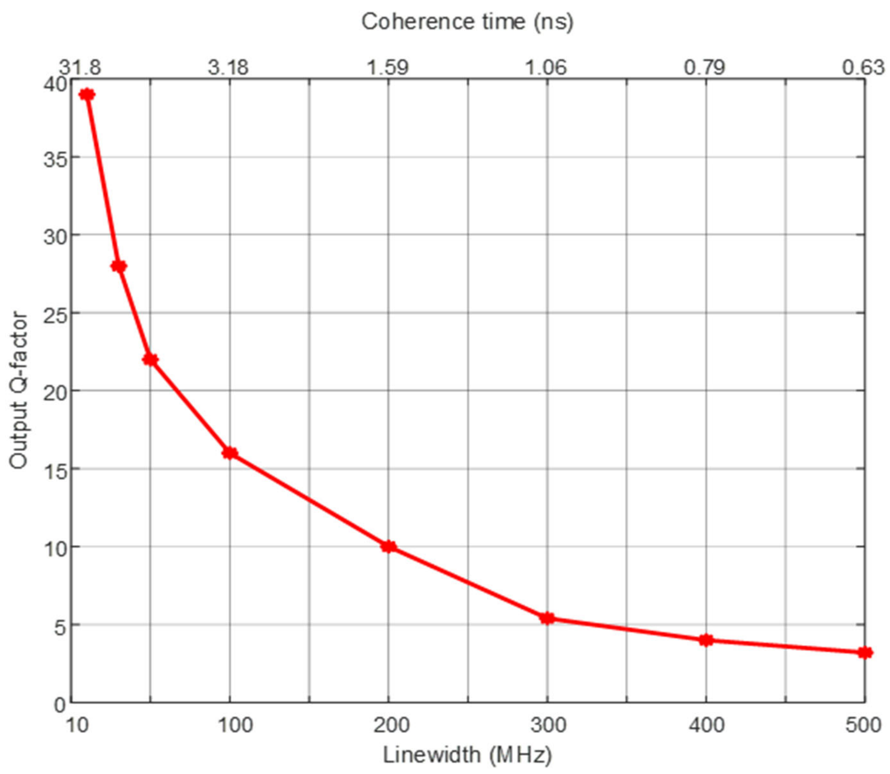

Supplementary Figure 5| Simulation results on the effect of laser coherence on NOT gate performance. Output signal quality-factor as a function of input signal spectral linewidth (inversely related to the coherence time) assuming an error-free input, 640 Gbit/s data rate and an 8 GHz linewidth spectral resolution from the Waveshaper phase filter (same parameters as those used for the simulation in Fig. 2).
